# SEOM guidelines 2014: improving quality to increase its usefulness

**DOI:** 10.1007/s12094-014-1246-y

**Published:** 2014-11-13

**Authors:** P. Garrido, C. A. Rodríguez

**Affiliations:** 1 Department of Medical Oncology, Spanish Society for Clinical Oncology, Hospital Universitario Ramon y Cajal, Madrid, Spain; 2 Department of Medical Oncology, Spanish Society for Clinical Oncology, Hospital Universitario de Salamanca-IBSAL, Salamanca, Spain

Since 2010, when the Spanish Society for Medical Oncology (SEOM) started publishing clinical practice guidelines for the management of solid tumours and supportive care, the challenges have evolved from educational purposes to policy purposes. Due to our increasingly complex system, patient care requires cohesive documents on which to base daily decisions [1–3] and SEOM is really committed to working on balancing both sustainability and equity in the access to new treatment options.

The aim of the guidelines was to combine updates of common tumours as well as to introduce new issues. This is one the reasons that explains their success as a consolidated project [4]. In the current edition, an independent external committee has been added to ensure the utmost rigour based on the level of evidence and grades of recommendations while maintaining their high practical value. There is no doubt that this decision will increase their value.

The nine guidelines to be published include highly relevant topics such as prevention and screening, hydroelectrolitic disorders, prostate cancer, management of thromboembolic events, renal cancer, gastroenteropancreatic neuroendocrine tumours, thyroid cancer, ovarian tumours and cancer of unknown origin.

While earlier diagnosis and advances in treatment have considerably improved survival in recent years, further progress is needed. In the light of the current status of knowledge, primary and secondary prevention of the most prevalent neoplasms (breast, cervix, prostate, colon and lung) are reviewed by SEOM experts. One of the greatest challenges is the fact that current therapies for advanced diseases are not curative, necessitating treatment over a number of years and placing a significant health care burden on society. To reduce this burden, care delivery must be more efficient and cost-effective. In particular, developments of adequate screening programs are needed, along with preventative strategies [5].

This is the first time that guidelines on hydroelectrolytic disorders have been included. We are aware of the importance acquired by some recent advances in this field and the need for comprehensive training in symptoms common to different tumours [6]. Similarly, a review of the management of thromboembolic events has also been considered. Venous thromboembolic events in oncological patients are very common and have specific challenges. These guidelines summarise the most recent recommendations about prophylaxis and treatment of these events, as well as help to address some doubts about specific clinical problems [7].

The options for patients with prostate cancer have recently increased with the addition of several new treatments that need to be analysed from a medical oncology perspective [8]. Similarly, the approaches to the available options for renal cancers have been updated [9].

Gastroenteropancreatic Neuroendocrine Neoplasms are a challenging family of tumours of growing incidence and varied clinical management. Significant advances in the diagnosis and the understanding of the molecular biology of these tumours have been achieved. Diagnostic workup, histological and staging classifications and the different available therapeutic approaches are briefly discussed in the manuscript from a practical point of view [10].

A further example of recent advances in the diagnosis, molecular biology and systemic therapeutics is thyroid cancer. This is the reason why new recommendations have been considered necessary [11]. Treatment options for the management of ovarian carcinoma have been also updated, including the most recent findings regarding their biology and new targeted therapies [12].

Last but not least, we still have the challenge of managing patients with “cancer of unknown origin”. This is a constantly changing area where a delicate balance is continuously needed. SEOM guidelines are intended to help oncologists in making their decisions [13].
